# Insights into the genetic variation profile of *tprK* in *Treponema pallidum* during the development of natural human syphilis infection

**DOI:** 10.1371/journal.pntd.0007621

**Published:** 2019-07-22

**Authors:** Dan Liu, Man-Li Tong, Yong Lin, Li-Li Liu, Li-Rong Lin, Tian-Ci Yang

**Affiliations:** 1 Center of Clinical Laboratory, Zhongshan Hospital, School of Medicine, Xiamen University, Xiamen, China; 2 Institute of Infectious Disease, School of Medicine, Xiamen University, Xiamen, China; University of Washington, UNITED STATES

## Abstract

**Background:**

Although the *tprK* gene of *Treponema pallidum* are thought to play a critical role in the pathogenesis of syphilis, the profile of variations in *tprK* during the development of human syphilis infection have remained unclear.

**Methods/Principal findings:**

Through next-generation sequencing, we compared the *tprK* gene of 14 secondary syphilis patients with that of 14 primary syphilis patients, and the results showed an increased number of variants within the seven V regions of the *tprK* gene in the secondary syphilis samples. The length of the sequences within each V region also presented a 3-bp changing pattern. Interestingly, the frequencies of predominant sequences within the V regions in the secondary syphilis samples were generally decreased compared with those found in the primary syphilis samples, particularly in the V7 region, where a frequency below 60% was found in up to 57% (8/14) of all secondary samples compared with 7% (1/14) of all primary samples. Moreover, the number of minor variants distributed between frequencies of 10 and 49.9% was increased. The alignment of all amino acid sequences within each V region of the primary and secondary syphilis samples revealed that some amino acid sequences, particularly the amino acid sequences IASDGGAIKH and IASEDGSAGNLKH in V1, were highly stable. Additionally, the amino acid sequences in V6 also exhibited notable intrastrain heterogeneity and were likely to form a strain-specific pattern at the interstrain level.

**Conclusions:**

The identification of different profiles of the *tprK* gene in primary and secondary syphilis patients indicated that the *tprK* gene of *T*. *pallidum* undergoes constant variation to result in the best adaptation to the host. The highly stable peptides found in V1 are likely promising potential vaccine components. The highly heterogenetic regions (e.g., V6) could help to understand the role of *tprK* in immune evasion.

## Author summary

Antigenic variation of the TprK antigen has been acknowledged to explain the persistence of *Treponema pallidum* in the host, however, the profile of variations in *tprK* during the development of human syphilis infection has not been well characterized. Here, we performed next-generation sequencing to compare the variations in *tprK* between primary and secondary syphilis samples. The profiles of *tprK* in the samples at different stages showed differences. A higher amount of pool variants within seven V regions was found in the secondary syphilis samples, and the frequencies of their predominant sequences generally decreased with increases in the number of minor variants with frequencies in the range of 10 to 49.9%. However, the length of variable sequences within the V regions of *tprK* in the secondary syphilis samples also presented a 3-bp changing pattern. Notably, the amino acid sequences IASDGGAIKH and IASEDGSAGNLKH in V1 not only presented a high proportion of interstrain sharing but also were found at a relatively high frequency (above 80%) in the populations. The sequences in V6 of the samples demonstrated substantial variability at the intra- and interstrain levels. These findings could provide insights into the potential syphilis vaccine components and the role of TprK in immune evasion.

## Introduction

Syphilis is a complex chronic disease caused by infection with *Treponema pallidum* subsp. *pallidum* (*T*. *pallidum*). Specifically, the disease has a series of highly distinct clinical stages [1], which usually includes the localized chancre primary stage, the disseminated secondary stage, and the late tertiary stage in untreated individuals [2]. This pattern of successive episodes during infection evokes other similar chronic diseases in which antigenic variation explains this characteristic long-term infection [3, 4]. Previous studies have indicated that antigenic variation in outer membrane antigens is a hallmark of many multistage infectious diseases [5–7].

Investigations of *tprK* from a 12-member gene family (*tpr*) of the *T*. *pallidum* have revealed that *tprK* is highly heterogeneous at both inter- and intrastrain levels. The sequence diversity of this gene is restricted to seven discrete variable (V) regions (V1–V7), which are separated by conserved sequences [8–10]. Although further investigations are needed to determine whether TprK is an outer membrane antigen [11–13], researchers have found that infection-induced antibody responses are directly related to V regions of TprK, where sequence variations would abrogate specific antibody binding [14, 15]. Therefore, it has been hypothesized that antigenic variations in TprK would facilitate *T*. *pallidum* to escape immune clearance and thereby allow the pathogen to persist in the host. Remarkable results using rabbit models support this hypothesis [14–16]. In our previous study [17], we employed a more sensitive and reliable approach, next-generation sequencing (NGS), to explore the *tprK* gene of *T*. *pallidum* directly from primary syphilis patient samples instead of rabbit-derived samples and found that variations in the V regions of *tprK* generally exhibited a sequence pool containing a high-proportion sequence (frequency above 80%) and many low-frequency minor variants (frequency below 20%). Only specific V region sequences appeared at frequencies of 20–80%.

Based on these findings, we were interested on the variations in the *tprK* gene in secondary syphilis samples. Comparisons of the variations between primary and secondary syphilis infection could provide notable information on the association of genetic variations in *tprK* with disease progression, help researchers gain insights into the processes underlying immune evasion by the pathogen and aid the identification of potential vaccine components for human immunology study.

## Materials and methods

### Ethics statement

The subjects included in this study were adults, and all of the subjects provided written informed consent in accordance with the institutional guidelines prior to the study. This study was approved by the Institutional Ethics Committee of Zhongshan Hospital, School of Medicine, Xiamen University, and complied with national legislations and the Declaration of Helsinki guidelines.

### Collection of clinical materials and isolation of DNA

25 skin lesion samples (erythema or condylomata lata) were collected from patients with secondary syphilis. The clinical diagnosis of syphilis was based on the US Centers for Disease Control and Prevention (CDC) [18] and the European CDC (ECDC) guidelines [19]. The lesions were placed into a sterile Petri dish containing 1 mL of saline (containing 20% normal rabbit serum), minced into very small pieces and squeezed into the liquid [20]. The samples were then examined by dark field microscopy, and the positive samples were used for subsequent DNA extraction.

DNA extraction was performed using the QIAamp DNA Mini Kit (Qiagen, Inc., Valencia, CA, USA) as previously described [21]. qPCR targeting *tp0574* was performed to determine whether each DNA sample contained treponemal DNA. 14 secondary syphilis samples (S-1~14) were ultimately included in this study. The samples were then subjected to molecular typing using the Enhance CDC system [22] and amplification of *tp0136* to determine whether they belonged to the Nichols-like group or SS14-like group [23].

### Segmented amplification of the *tprK* gene

Segmented amplification of the *tprK* gene was conducted as described previously [17]. Briefly, the extracted DNA was directly used for amplification of the *tprK* gene open reading frame (ORF), and the amplicons were gel purified. The purified *tprK* amplicons (diluted 100×) were used as the segmented amplification template for partial amplification of four fragments of 400–500 bp (overlapping by at least 20 bp) covering the *tprK* ORF. All the products were verified by 2% agarose gel electrophoresis and gel purified. A high-fidelity PCR polymerase, KOD FX Neo polymerase (Toyobo, Osaka, Japan), was used for the amplification, and the amplification primers are shown in S1 Table. The four subfragment amplicons corresponding to each sample were mixed in equimolar amounts into one pool to produce a separate library, and a barcode was used to distinguish each sample.

### Library construction and next-generation sequencing

Library construction and sequencing were performed by Sangon Biotech Company (Shanghai, China) using the MiSeq platform (Illumina, San Diego, CA, USA) in the paired-end sequencing (2×300 bp) mode. The FastQC and FASTX tools were applied to check and improve the quality of the raw sequence data, respectively. The final reads of *tprK* were compared with the *tprK* of the Seattle Nichols strain (GenBank Accession Number AF194369.1) using Bowtie 2 (version 2.1.0) to estimate the sequencing depth and coverage.

### Evaluation of intrastrain heterogeneity of the *tprK* gene variable regions (V1-V7)

Based on a previously described principle for the extraction of sequence data [17], an in-house Perl script was applied to specifically capture DNA sequences within seven regions of the *tprK* gene from the raw data, both forward and reverse. Thus, the exact number of distinct sequences within seven variable regions of the *tprK* gene from each sample was acquired. The intrastrain heterogeneous sequences were valid if the following conditions were simultaneously verified: (1) supported by at least fifty reads and (2) with a frequency above 1%. The relative frequency of the sequences within each variable region was then calculated. To systemically present the variation characteristics of *tprK* at different clinical stages, we included previous data for *tprK* in primary syphilis patients (X-1~14) for comparison purposes [17].

### Statistical analysis

All statistical analyses were performed using SPSS version 22.0 (SPSS, Chicago, IL, USA). To compare the frequencies below 60% in V7 in secondary versus primary samples, odds ratios were estimated by logistic regression. The Chi-square test was used to identify differences in the amount of variants captured in seven variable regions of the *tprK* gene between primary syphilis samples and secondary syphilis samples, and the distribution of minor variants between the samples at two different stages comprised three ranges (1–5%, 5–10% and 10%-49.9%). A two-sided *P* value < 0.05 was considered statistically significant.

### Accession numbers

The raw data of *tprK* obtained in this study were deposited in the SRA database (BioProject ID: PRJNA512914) under the following BioSample accession numbers: SAMN10690826- SAMN10690839 for S-1~14. The data of *tprK* in previous studies were deposited in the SRA database (BioProject ID: PRJNA498982) under the following BioSample accession numbers: SAMN10340238-SAMN10340251 for X-1~14.

## Results

### Background data for *tprK* in secondary syphilis patients obtained by NGS

The 14 secondary syphilis samples (S-1~14) were collected at Zhongshan Hospital, Xiamen University. The clinical information for all 14 patients is shown in Table 1. The data obtained from the qPCR analysis of the *tp0574* gene showed that each DNA sample contained a certain amount of treponemal DNA for amplification of full-length *tprK*. Molecular typing using the ECDC system detected seven different genotypes, and genotype 16d/f was the most prevalent in these 14 samples (S2 Table). Based on the sequencing data for the *tp0136* gene, most strains belonged to the SS14-like group, and only three strains (S-7, S-9 and S-12 strains) belonged to the Nichols-like group. The median sequencing depth of the *tprK* segment samples ranged from 9810.91 to 52366.84, and the coverage ranged from 99.34% to 99.61%, indicating high identity with the *tprK* gene of the Seattle Nichols strain (S2 Table). To more clearly present the results, background data for the 14 primary syphilis samples (X-1~14) obtained in previous studies were also included in Table 1.

**Table 1:** Clinical characteristics of the study participants.

Characteristic	Clinical stage	
	Primary (n = 14)*	Secondary (n = 14)
**Gender**		
Male n (%)	13 (92.9)	10 (71.4)
Female n (%)	1 (7.1)	4 (40.0)
Age (Mean±SD)	53.9±13.7	39.7±15.7
Serum RPR titer, Median (IQR)	1:16 (1:4–1:32)	1:32 (1:16–1:128)
Serum TPPA	Positive	Positive
Dark field microscopy	Positive	Positive
**Clinical manifestations**		
Chancre	13	0
Lymphadenopathy	1	0
Condyloma	0	11
Erythema	0	3
**Genetic group by *tp0136***		
Nichols-like group	2	3
SS14-like group	12	11

Abbreviations: RPR, reactive plasma reagin; TPPA, *T*. *pallidum* particle agglutination; IQR, interquartile range.

* the data were previously published [17].

### Characteristic profile of *tprK* in secondary syphilis samples

**Fig 1:**
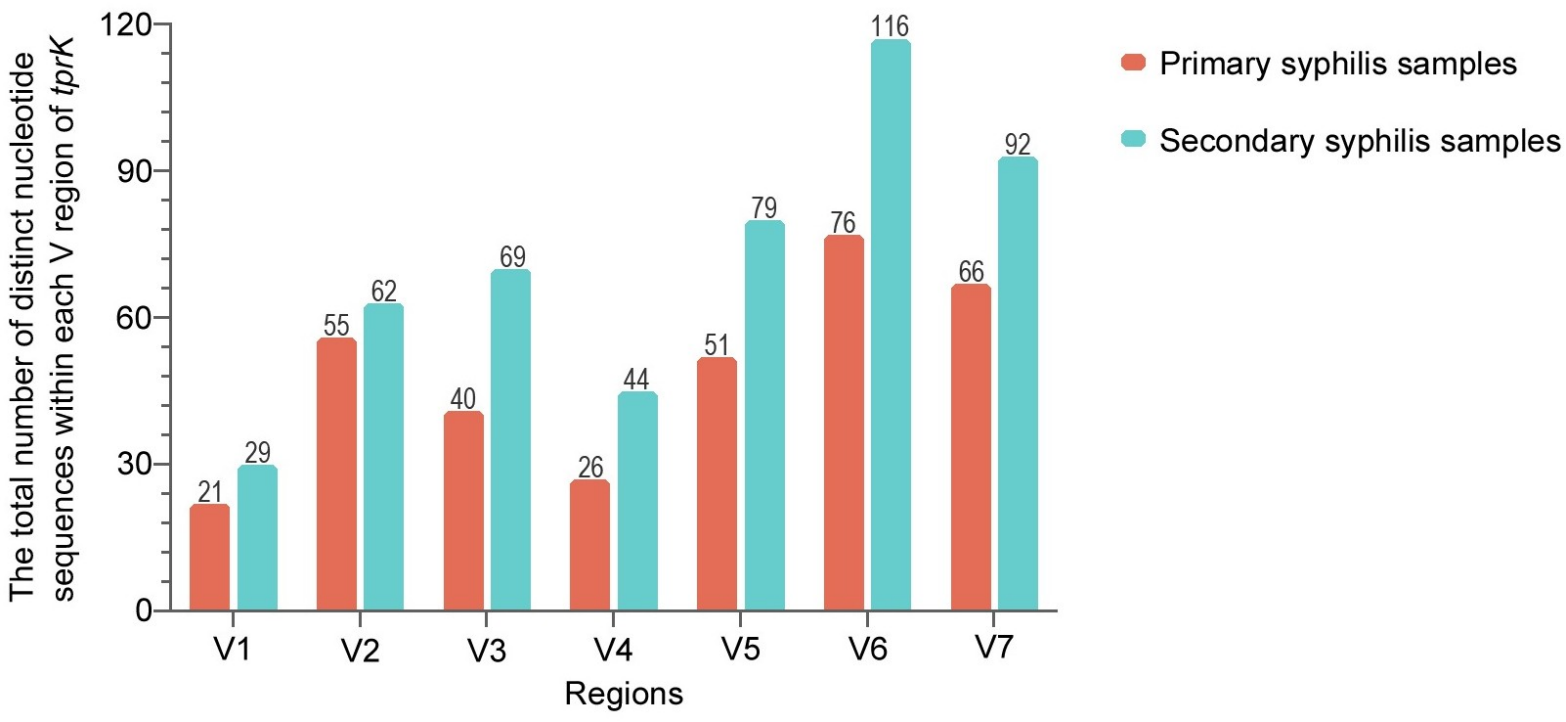
A sum of distinct nucleotide sequences within each V region of *tprK* from each strain in the samples at different stages. The data of *tprK* from primary syphilis samples were obtained in previous studies [17].

**Fig 2:**
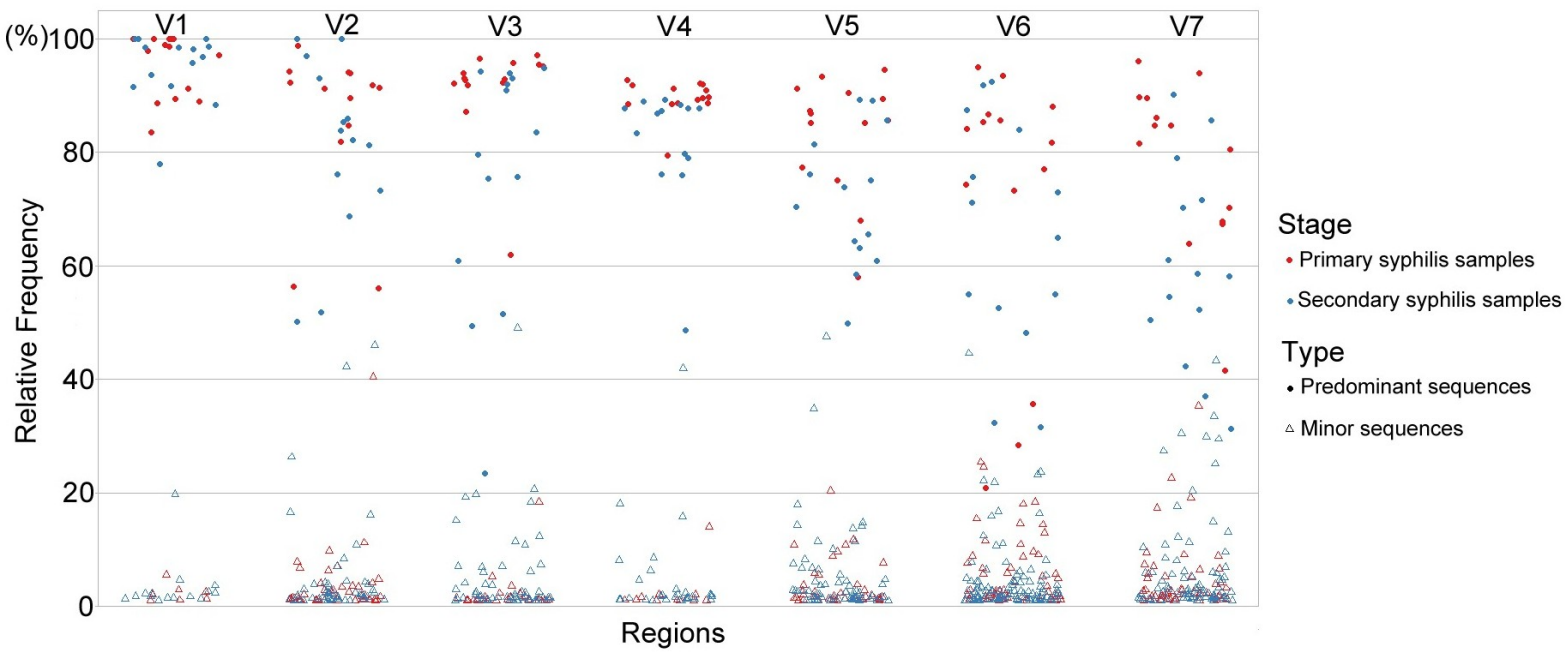
Proportional distribution of the captured distinct nucleotide sequences within each V region of *tprK* from each strain. Each circle presents the relative frequency of a distinct sequence within the V region in each strain. The solid and hollow circles indicate the predominant sequences and the minor variants in the populations, respectively. The two colors specify the strains from the samples at the two different stages.

Using the extraction strategy, distinct nucleotide sequences in the individual V regions of *tprK* were captured from each sample, and 491 sequences were obtained for the 14 secondary syphilis samples (Fig 1). Calculation of the relative frequencies of distinct sequences within each V region in a single strain revealed that the *tprK* gene in secondary syphilis samples also contained a predominant sequence within the regions (Fig 2). However, the distribution of the sequences within seven V regions presented some dispersity: the predominant variants had broader frequency spectra (almost between 20–80%), and the minor variants reached higher frequencies (above 20%).

### Comparison of the profiles of *tprK* in the context of primary and secondary infection

Compared with the sequences captured within each V region from primary syphilis samples in previous studies (335 in total) (S3 Table), the secondary syphilis samples presented a higher number of variants within each V region. The Chi-square test was used to identify differences in the amounts of variants captured within each V region of the *tprK* gene. The trend found for the sequence variability in the V regions of *tprK* showed no significant differences between the samples at the two different stages (*P* = 0.767), and the highest and lowest sequence variability was found in V6 and V1, respectively (Fig 1). Compared to the frequencies of predominant sequences (frequency almost above 80%) within the V regions among primary syphilis samples, the frequencies of the sequences within the V regions among secondary syphilis samples were generally lower and this finding was particularly true for the V7 region, where a frequency below 60% was found in up to 57% (8/14) of the secondary samples compared with 7% (1/14) of the primary samples. We used logistic regression to estimate the odds radio for frequencies below 60% appearing in V7 among secondary versus primary samples. The odds ratio for frequencies below 60% appearing in V7 among secondary samples were 17.3-fold higher than those found among primary samples (OR = 17.3 [95% confidence interval, 1.75 to 171.78]; *P* = 0.015). Notably, the frequencies of predominant sequences in V1 among all 28 samples remained almost above 80%.

**Fig 3:**
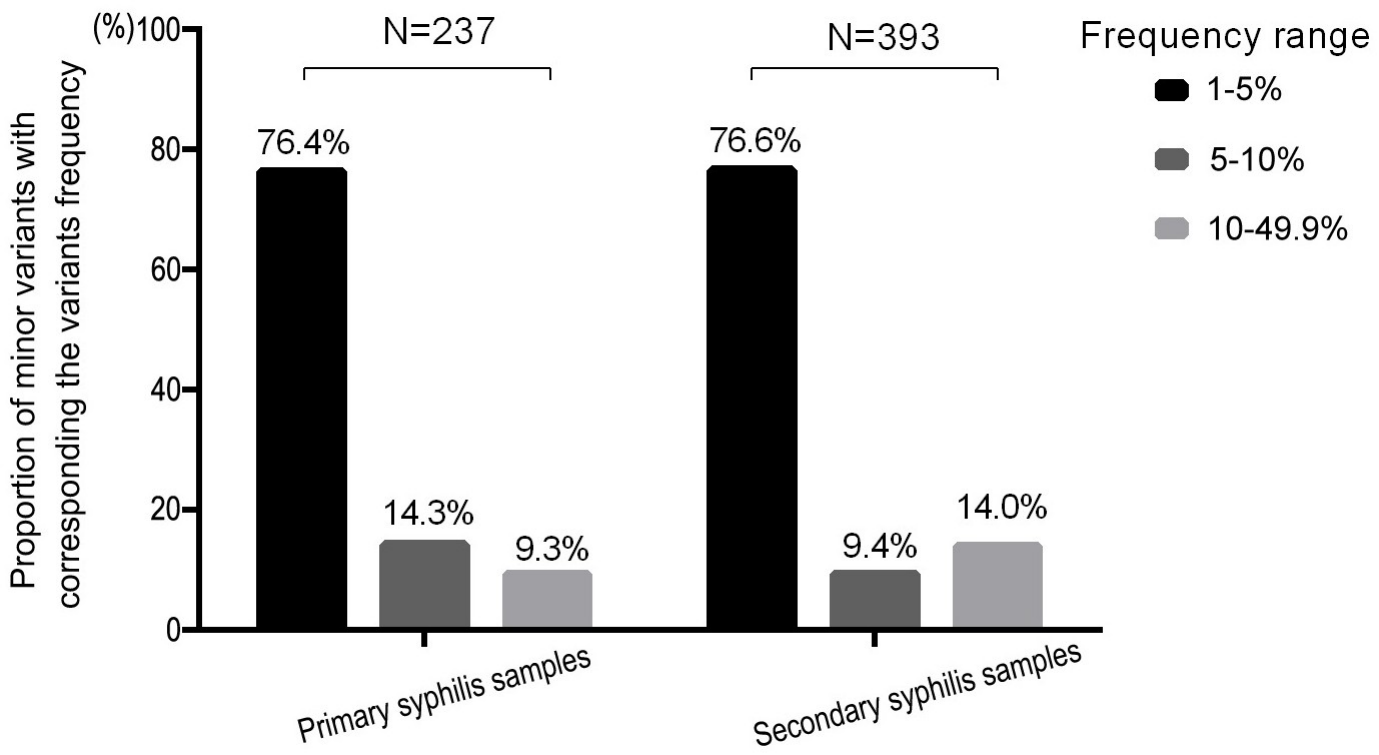
Proportional distribution of minor variants in three frequency ranges (1–5%, 5–10% and 10–49.9%). N represents the total amount of minor variants detected in the primary and secondary syphilis samples. The proportional distributed in the three frequency ranges indicated the number of variants within each range relative to the total amount of minor variants.

In the secondary syphilis samples, *tprK* still contained a pool of minor variants within each V region. As shown in Fig 3, most of the minor variants were concentrated in the frequency range of 1–5% in both groups, and the proportions in the other two frequency ranges (5–10% and 10–49.9%) among the secondary syphilis samples were reversed relative to the distribution pattern in primary syphilis samples (9.4% and 14.0%, 14.3% and 9.3%, respectively). However, the Chi-square test was used to investigate the distribution of minor variants in these three ranges, and no significant difference was found between the primary and secondary syphilis samples (*P* = 0.053).

Additionally, the length of variable sequences within the V regions in secondary syphilis samples corroborated the finding that the length of these variants within the V regions differed in multiples of 3 bp (S4 Table). Compared with the lengths within the regions in primary syphilis samples, V3 and V5 in the secondary syphilis samples maintained the same forms, but the other regions showed the appearance of some new lengths (V1, V4, V6 and V7) or the disappearance of some lengths (V2 and V7).

### Amino acid sequence within each V region of the *tprK* gene

We translated the variable nucleotide sequences within each V region *in silico*. No early terminations or changes in the reading frames in *tprK* were found among the secondary samples, and synonymous sequences were rare and also only found in V2 and V5 (S5 Table). Similar to the phenomenon found among the primary syphilis samples, substantial interstrain sequence redundancy was found in each V region. Altogether, V1, V2 and V4 showed strong shared sequence ability, and V6 showed the least shared ability region (Table 2).

**Table 2:** Interstrain redundancy in amino acid sequences within seven V regions of the *tprK* gene in the primary and secondary samples.

	Primary syphilis*			Secondary syphilis		
V regions	A sum of distinct sequences in single strain from 14 samples	No. case of synonymous mutations	Total sequences unique to 14 samples (%)	A sum of distinct sequences in single strain from 14 samples	No. case of synonymous mutations	Total sequences unique to 14 samples (%)
V1	21	0	11 (52.4)	29	0	14 (48.3)
V2	55	6	25 (45.5)	62	8	23 (37.1)
V3	40	0	34 (85.0)	69	0	54 (78.3)
V4	26	0	9 (34.6)	44	0	14 (31.8)
V5	51	2	31 (60.8)	79	4	46 (58.2)
V6	76	0	71 (93.4)	116	0	110 (94.8)
V7	66	0	55 (83.3)	92	0	66 (73.9)

* the data were previously published [17].

**Fig 4:**
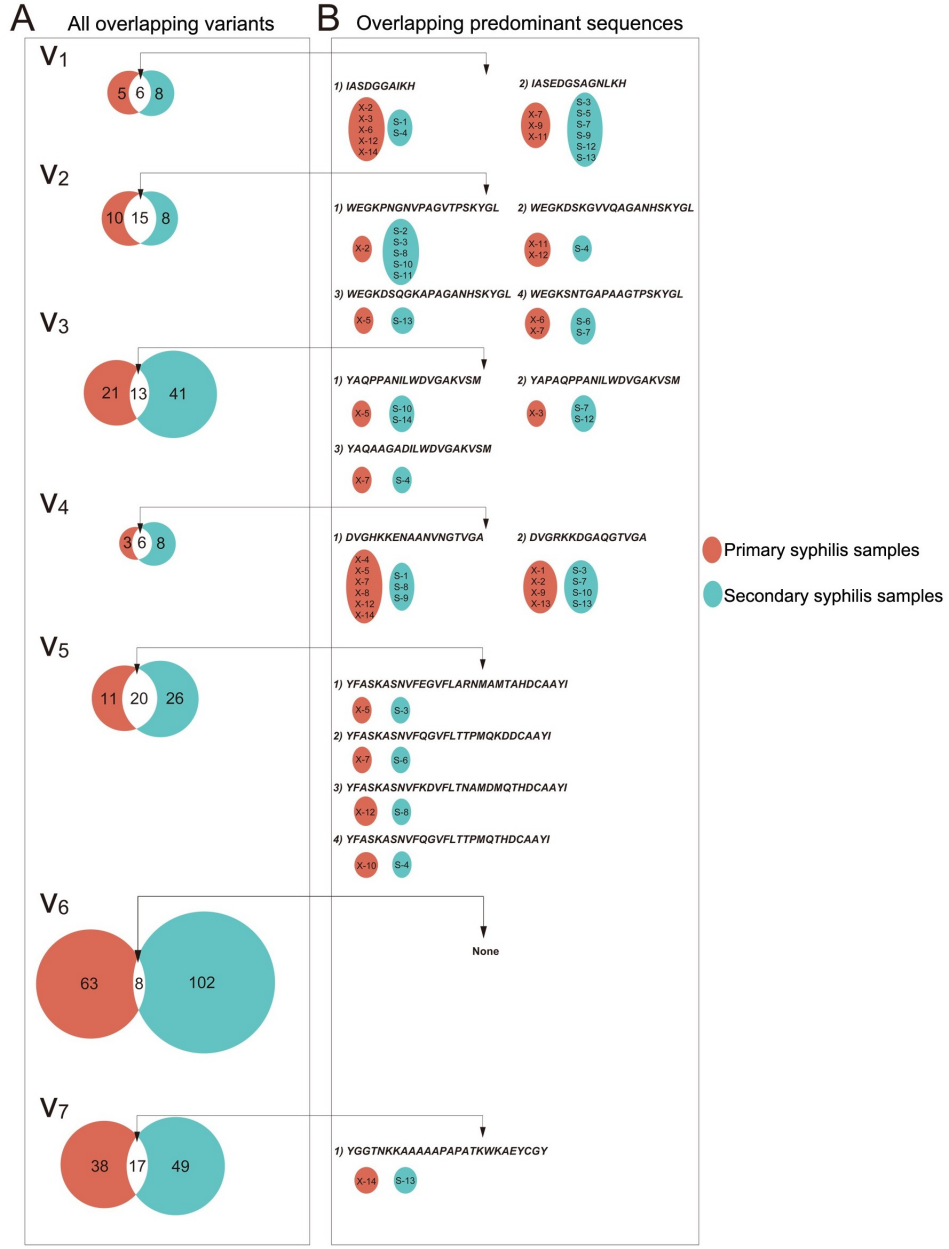
Overlapping amino acid sequences within the V region between the samples at the two different stages. (A) Each Venn diagram presents the overlapping amino acid sequences in the V regions of *tprK* in primary and secondary syphilis patients. The numbers in the red and green circles represent the total number of amino acid variants that are unique to primary and secondary syphilis patients, respectively. The number in the intersection of the two circles represents the number of overlapping variants. (B) The figure shows the overlapping predominant sequences within the V regions and the redundancy of these sequences in primary and secondary syphilis samples.

Furthermore, we determined whether a specific V region sequence found in the secondary syphilis samples also presented in the primary syphilis samples. After aligning the amino acid sequences that were unique to the samples at one of the two stages, we found that a number of sequences in V1, V2 and V4 that were specific to the secondary samples were also found in the primary samples (Fig 4A). Notably, the predominant sequences of the V regions also presented overlapping (Fig 4B). Among the seven V regions, V2 and V5 showed more identical predominant sequences between samples at the two different stages. However, the sequences were only found in a few samples. The analysis of the sequences in V1 and V4 showed that although V1 and V4 only presented two identical predominant sequences (IASDGGAIKH and IASEDGSAGNLKH in V1 and DVGHKKENAANVNGTVGA and DVGRKKDGAQGTVGA in V4), the identical sequences showed high interstrain sharing. In addition, the frequencies of the two shared sequences in V1 reached 80% in the strains.

As previously described, V6 was the most variable region of *tprK*. We further corroborated this feature in the context of primary and secondary syphilis infection. As shown in Fig 4A, only eight identical sequences were found in the samples at the two different stages, and the proportions of these overlapping sequences relative to the unique sequences in the primary and secondary samples were 12.7% (8/71) and 7.8% (8/110), respectively. Moreover, none of the predominant sequences were identical (Fig 4B). The levels of nucleotide diversity in V6 between each sample (Dxy) were calculated using DnaSP v.6.12.01. The Dxy nucleotide diversity in V6 across each sample was almost above 0.15 (S1 Fig), which was in agreement with the proposed view that V6 presents high diversity among most *T*. *pallidum* strains.

## Discussion

With the identification of a 12-member gene family (*tpr*) in the Nichols strain of *T*. *pallidum* [24], the antigen-coding *tprK* had been extensively studied because of its highly variable antigenic profile [9, 10, 14, 25, 26]. Similar to known mechanisms through which many pathogens undergo antigenic variation to evade the immune system and establish chronic infection in the host [5, 27], *tprK* is believed to play an essential role in the pathogenesis of syphilis [15, 16]. Hence, efforts to understand *tprK* diversity in the context of human infection, particularly at different clinical stages, would be beneficial to the clinical elucidation of the role of *tprK* in successive episodes of this chronic infection and would contribute to a more in-depth understanding of the pathogenesis of syphilis.

In this study, NGS was used in combination with an in-house Perl script to confirm the features characterizing the diversity of the *tprK* gene during natural human infection: *tprK* contained a predominant sequence and numerous minor variants within each V region, and most variants were found at low frequency in the range of 1 to 5%. Interestingly, in primary syphilis samples, the frequencies of predominant variants were almost above 80%, and those of minor variants were almost below 20%. However, the predominant variants in secondary syphilis samples had broader frequency spectra (almost between 20 and 80%), and more minor variants reached higher frequencies with a broader range of frequencies (above 20%). Combining these two different profiles of the *tprK* gene, it seems that the variants within the V regions in secondary syphilis samples fill in the middle zone which is almost empty in primary syphilis pattern. This finding suggested that the variations in the *tprK* gene might follow a logical fitness-based evolution. An analysis of *T*. *pallidum* infection at the primary stage showed that the sequences within each V region of the *tprK* gene presented a two-level distribution (above 80% and below 20%), suggesting the high frequency sequences may be better associated with the avoidance of immune recognition. Changes in the immune environment (development into secondary syphilis infection) could cause the original fitted sequences within each V region to no longer facilitate the survival of *T*. *pallidum*. The original predominant sequences in V regions need to change to obtain a new better TprK epitope for *T*. *pallidum*. At present, the frequencies of the predominant sequences are lower in the populations, and certain minor variants might be selected and exhibit higher frequencies in the populations. As a result, the original sequences might disappear, and new advantageous sequences would emerge [17, 28]. Due to the continuous evolution of the sequences of *tprK*, the TprK antigen in the infection process becomes increasingly diverse, which would enable *T*. *pallidum* to successively evade the antibody response and thereby establish chronic infection [15, 26].

In addition, we demonstrated that V6 might be the first region to change in primary syphilis samples [17]. In this study, we noted that the predominant sequences in V7 among secondary syphilis samples appeared at frequencies almost below 60% (*P* = 0.015), which might suggest that variations in V7 evolved following V6 and that the region might be important for the development of secondary syphilis infection [29]. Additionally, a strict 3-bp changing pattern in each variable region was further confirmed in the secondary syphilis samples, and no frame shifts have been found [9, 10, 29], which demonstrates the existence of an elaborate system for the regulation of *tprK* sequence variation.

Substantial interstrain sequence redundancy was observed in *tprK* among the samples at the two different stages. Among all V regions, the amino sequences IASDGGAIKH and IASEDGSAGNLKH in V1 and the amino sequences DVGHKKENAANVNGTVGA and DVGRKKDGAQGTVGA in V4 showed strong interstrain sharing ability across 28 clinical strains. Moreover, the sequences in V1 were those that presented a relatively high frequency (above 80%) in the populations. In fact, the two sequences were also found to be the most stable amino acid sequences among the samples in the investigation of Pinto *et al*. [29]. As described previously [17], a high-frequency amino acid sequence for antigen-coding *tprK* highly reflects the immune response of the host. Moreover, this sequence presented high-level interstrain sharing, that is, the sequence was found in several syphilitic patients, which indicates that TprK has a better-fitted epitope profile for allowing *T*. *pallidum* to adapt to its host. Among the seven V regions in *tprK*, V1 was found to be relatively stable among the samples at the two different stages, and the two sequences of V1 were most stable among the 28 clinical strains, which suggested that maintaining V1 relatively stable would be essential for the pathogen and that the stable peptides in V1 would be a promising vaccine component for future research [30]. Notably, the promising vaccine peptides found in this study might target the majority but not all of the strains. This problem should be considered further to explore the function of these peptides.

Additionally, we confirmed high heterogeneity at the intrastrain level in V6 throughout the infection process. The existence of a highly diverse region in this antigen-coding gene of an isolate of *T*. *pallidum* might greatly enable the pathogen to resist binding by existing opsonic antibodies and might make the pathogen less likely to be recognized by activated macrophages [15]. The amino acid sequences of V6 also presented high diversity at the interstrain level, showing a strain-specific pattern for the sequences, which might explain why the protection of TprK was compromised and a lack of heterologous protection [26]. Currently, it is very difficult to distinguish between treatment failure (relapse) and reinfection in clinical practice. Myint *et al*. [31] used a molecular method by analyzing *tprK* sequences to distinguish relapse from reinfection in a patient with recurrent secondary syphilis. Based on the results, whether there is speculation that the sequences in V6 retain a high homology in a relapse case, but the sequences are highly strain-specific in a reinfection case? This speculation requires further supporting evidence from additional future experiments.

Finally, the limitations of our study should be discussed. First, the limited sample size did not support us to draw further definitive conclusions, and the study did not explore the function of the sequences within each V region. A future study could investigate the different peptides in *tprK* regions observed between primary and secondary syphilis to explore the potential importance of these differences in host interactions and immune evasion. Moreover, the potential promising vaccine components identified in this study could be synthesizes to investigate the immune function of these peptides and thereby lay a foundation for vaccine development. Second, the study provided information on individual V regions instead of information on a single *tprK* ORF. Using a novel PacBio sequencing pipeline to obtain full length of the *tprK* sequence could be optimal. And the data might provide useful insights into the structure and function of TprK. Third, because the samples used in this study were from one lesion rather than different lesions, we cannot completely exclude the possibility that the individual patient was infected with different strains resulting in the initial diversity in *tprK*, even though we verified that the samples presented a single genetic background, as demonstrated by molecular typing (ECDC system and sequencing of *tp0136* locus). Additionally, the same genetic background of the tested samples may require similar studies to explore the potential relationship between the genetic background and the variations in *tprK*.

In this study, we revealed that the characteristic profiles of *tprK* in the context of primary and secondary infection were different, which indicated that throughout the development of the disease, *T*. *pallidum* might constantly undergo variations in its *tprK* gene to achieve its best adaptation to the host. Interestingly, *tprK* maintains a contradictory scenario during the course of infection, that is, having a relatively conserved region (V1) and a highly diverse region (V6). The stable sequences in V1 and the highly heterogeneous sequences in V6 could provide important information for exploring promising potential vaccine components and the role of *tprK* in persistent syphilis infection.

## Supporting information

S1 Fig Diversity of nucleotide sequences in V6 at the interstrain level. Only one nucleotide sequence was captured in the X-7 strain, and thus, no sequence diversity data were obtained for the X-7 strain.
(TIF)

S1 Table Primers for *tprK* amplification. (DOCX)

S2 Table Characteristics of clinical samples and sequencing background data of the *tprK* gene by NGS. (DOCX)

S3 Table Nucleotide sequences within the seven variable regions (V1-V7) of *tprK* captured from 28 clinical samples. The captured nucleotide sequences were ranked according to their relative frequency within each population of (A) 14 primary syphilis samples (X-1~14) in previous studies and (B) 14 secondary syphilis samples (S-1~14).
(XLSX)

S4 Table Length of distinct nucleotide sequences within seven V regions in *tprK* between primary and secondary samples. The numbers in the shaded V region lines represent the total number of distinct nucleotide sequences in that V region.
(DOCX)

S5 Table Amino acid sequences within the seven variable regions (V1-V7) of *tprK* captured from 28 clinical samples. The amino acid sequences from (A) 14 primary syphilis samples (X-1~14) in previous studies and (B) 14 secondary syphilis samples (S-1~14) are shown. The asterisk (*) indicates synonymous nucleotide sequences within the same strain.
(XLSX)
