# Supplementary figures and images for: Insights into the genetic variation profile of tprK in Treponema pallidum during the development of natural human syphilis infection
Source: PLoS Negl Trop Dis. 2019 Jul 22;13(7):e0007621. doi: 10.1371/journal.pntd.0007621 (PMC6675121; doi:10.1371/journal.pntd.0007621)

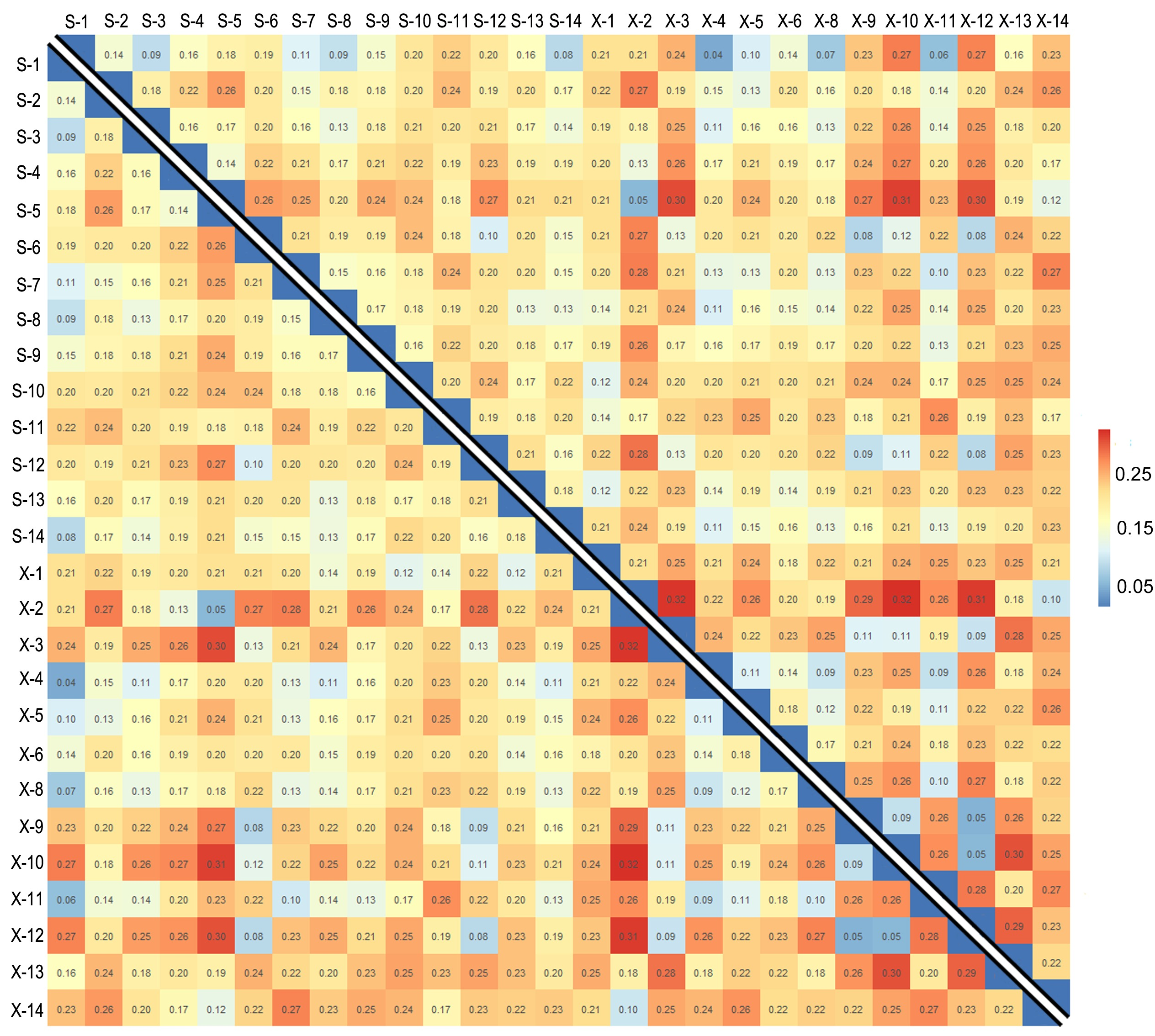

Supplement: S1 Fig — Only one nucleotide sequence was captured in the X-7 strain, and thus, no sequence diversity data were obtained for the X-7 strain. (TIF) [file pntd.0007621.s001.tif]
