# Supplementary material for: Insights into the genetic variation profile of tprK in Treponema pallidum during the development of natural human syphilis infection
Source: PLoS Negl Trop Dis. 2019 Jul 22;13(7):e0007621. doi: 10.1371/journal.pntd.0007621 (PMC6675121; doi:10.1371/journal.pntd.0007621)
Supplement: S2 Table — (DOCX) [file pntd.0007621.s003.docx]

**Supplementary Table 2. Characteristics of clinical samples and sequencing background data of the *tprK* gene by NGS**

| Strain | Gender | Age (year) | Serum RPR | Serum TPPA | Dark field microscopy | *T. pallidum* genome copies by *tp0574* | Genetic group by *tp*0136 | ECDCT | Total reads | On-target reads (%) | Mean depth of coverage |
| --- | --- | --- | --- | --- | --- | --- | --- | --- | --- | --- | --- |
| X-1 | Male | 45 | 1:16 | + | Positive | 8.2E+03 | SS14-like group | 14d/f | 357382 | 99.41 | 51967.28 |
| X-2 | Male | 27 | 1:16 | + | Positive | 8.82E+04 | Nichols-like group | 11d/c | 340240 | 99.47 | 49660.18 |
| X-3 | Male | 62 | 1:16 | + | Positive | 4.55E+04 | SS14-like group | 13d/f | 398898 | 99.41 | 56676.38 |
| X-4 | Male | 65 | 1:4 | + | Positive | 1.15E+04 | SS14-like group | 11d/f | 365060 | 99.34 | 52742.09 |
| X-5 | Male | 76 | 1:16 | + | Positive | 5.73E+04 | SS14-like group | 13d/f | 363940 | 99.61 | 52960.83 |
| X-6 | Male | 64 | 1:32 | + | Positive | 2.33E+02 | SS14-like group | 16d/f | 106934 | 99.37 | 14249.15 |
| X-7 | Female | 56 | 1:16 | + | Positive | 1.26E+04 | Nichols-like group | 22d/c | 114012 | 99.37 | 15579.12 |
| X-8 | Male | 46 | 1:4 | + | Positive | 1.41E+04 | SS14-like group | 15d/f | 103280 | 99.43 | 12951.11 |
| X-9 | Male | 40 | 1:4 | + | Positive | 1.39E+03 | SS14-like group | 16d/f | 119552 | 99.43 | 15864.28 |
| X-10 | Male | 66 | 1:32 | + | Positive | 9.17E+03 | SS14-like group | 16d/f | 114064 | 99.37 | 14927.08 |
| X-11 | Male | 44 | 1:2 | + | Positive | 2.67E+02 | SS14-like group | 16d/f | 94572 | 99.50 | 12935.89 |
| X-12 | Male | 39 | - | + | Positive | 6.40E+03 | SS14-like group | 16d/f | 114588 | 99.43 | 14944.66 |
| X-13 | Male | 63 | 1:16 | + | Positive | 2.02E+02 | SS14-like group | 15d/f | 118634 | 99.37 | 15013.54 |
| X-14 | Male | 61 | 1:1 | + | Positive | 1.16E+03 | SS14-like group | 17d/f | 82812 | 99.37 | 10568.99 |
| S-1 | Female | 30 | 1:32 | + | Positive | 4.42E+03 | SS14-like group | 15d/f | 363128 | 99.34 | 52366.84 |
| S-2 | Male | 58 | 1:32 | + | Positive | 2.24E+04 | SS14-like group | 11d/f | 354046 | 99.34 | 51107.41 |
| S-3 | Male | 67 | 1:128 | + | Positive | 1.90E+03 | SS14-like group | 16d/f | 107952 | 99.37 | 14253.29 |
| S-4 | Male | 42 | 1:16 | + | Positive | 1.41E+03 | SS14-like group | 16d/f | 114314 | 99.43 | 14328.57 |
| S-5 | Male | 28 | 1:16 | + | Positive | 9.06E+02 | SS14-like group | 17d/f | 117932 | 99.37 | 15440.63 |
| S-6 | Female | 32 | 1:128 | + | Positive | 2.65E+02 | SS14-like group | 16d/f | 91080 | 99.37 | 11911.52 |
| S-7 | Male | 35 | 1:16 | + | Positive | 2.20E+03 | Nichols-like group | 23d/c | 114584 | 99.50 | 13301.06 |
| S-8 | Female | 36 | 1:16 | + | Positive | 1.05E+04 | SS14-like group | 16d/f | 80226 | 99.37 | 10367.92 |
| S-9 | Male | 22 | 1:32 | + | Positive | 1.03E+04 | Nichols-like group | 22d/c | 103868 | 99.43 | 13907.47 |
| S-10 | Male | 25 | 1:32 | + | Positive | 4.01E+04 | SS14-like group | 15d/f | 151990 | 99.37 | 19886.38 |
| S-11 | Female | 41 | 1:32 | + | Positive | 4.00E+02 | SS14-like group | 16d/f | 107022 | 99.43 | 14192.04 |
| S-12 | Male | 20 | 1:32 | + | Positive | 1.84E+04 | Nichols-like group | 21d/c | 126090 | 99.43 | 17031.70 |
| S-13 | Male | 65 | 1:128 | + | Positive | 8.57E+03 | SS14-like group | 15d/f | 74684 | 99.37 | 9810.91 |
| S-14 | Male | 55 | 1:32 | + | Positive | 1.36E+02 | SS14-like group | 16d/f | 93818 | 99.37 | 12129.14 |

Abbreviations: NGS, next generation sequencing; RPR, reactive plasma reagin; TPPA, *T. pallidum* particle agglutination; ECDCT, enhanced CDC-typing +, positive;-, negative.
