# Supplementary material for: Insights into the genetic variation profile of tprK in Treponema pallidum during the development of natural human syphilis infection
Source: PLoS Negl Trop Dis. 2019 Jul 22;13(7):e0007621. doi: 10.1371/journal.pntd.0007621 (PMC6675121; doi:10.1371/journal.pntd.0007621)
Supplement: S4 Table — The numbers in the shaded V region lines represent the total number of distinct nucleotide sequences in that V region. (DOCX) [file pntd.0007621.s005.docx]

**Supplementary Table 4. Length of distinct nucleotide sequences within seven V regions in *tprK* between primary and secondary syphilis samples**

| **Region** | **Length (bp)** | **Percent (%)** | |
| --- | --- | --- | --- |
|  |  | **Primary*** | **Secondary** |
| **V1** |  | **21** | **29** |
|  | 32 | 28.6 | 10.3 |
|  | 35 | - | 6.9 |
|  | 38 | 14.3 | 6.9 |
|  | 41 | 57.1 | 72.4 |
|  | 44 | - | 3.4 |
| **V2** |  | **55** | **62** |
|  | 59 | 5.5 | 8.1 |
|  | 62 | 50.9 | 59.7 |
|  | 65 | 41.8 | 32.2 |
|  | 68 | 1.8 | - |
| **V3** |  | **40** | **69** |
|  | 54 | 2.5 | 10.1 |
|  | 57 | 7.5 | 10.1 |
|  | 60 | 25.0 | 20.3 |
|  | 63 | 22.5 | 14.5 |
|  | 66 | 7.5 | 8.7 |
|  | 69 | 10.0 | 4.4 |
|  | 72 | 2.5 | 4.4 |
|  | 75 | 12.5 | 13.0 |
|  | 78 | 2.5 | 8.7 |
|  | 81 | 5.0 | 4.4 |
|  | 84 | 2.5 | 1.4 |
| **V4** |  | **26** | **44** |
|  | 49 | 57.7 | 59.1 |
|  | 52 | - | 18.2 |
|  | 55 | 7.7 | 2.3 |
|  | 58 | 34.6 | 20.4 |
| **V5** |  | **51** | **79** |
|  | 84 | 72.5 | 65.8 |
|  | 90 | 27.5 | 34.2 |
| **V6** |  | **76** | **116** |
|  | 42 | - | 1.7 |
|  | 45 | 5.3 | 2.6 |
|  | 48 | 3.9 | 10.3 |
|  | 51 | 10.5 | 4.3 |
|  | 54 | 9.2 | 4.3 |
|  | 57 | 11.8 | 14.7 |
|  | 60 | 17.1 | 10.3 |
|  | 63 | 14.5 | 12.9 |
|  | 66 | 10.5 | 11.2 |
|  | 69 | 5.3 | 11.2 |
|  | 72 | 7.9 | 5.2 |
|  | 75 | 1.3 | 5.2 |
|  | 78 | 2.6 | 0.9 |
|  | 81 | - | 3.4 |
|  | 84 | - | 1.7 |
| **V7** |  | **66** | **92** |
|  | 64 | 6.1 | 1.1 |
|  | 67 | 1.5 | 9.8 |
|  | 70 | 9.1 | 16.3 |
|  | 73 | 9.1 | 10.8 |
|  | 76 | 6.1 | 7.6 |
|  | 79 | 16.7 | 16.3 |
|  | 82 | 9.1 | 18.5 |
|  | 85 | 25.7 | 14.1 |
|  | 88 | 10.6 | 2.2 |
|  | 91 | 4.5 | - |
|  | 94 | 1.5 | - |
|  | 100 | - | 3.3 |

*the data were previously published [17].

The numbers in the shaded V region lines represent total number of different nucleotide sequences seen in that V region within each sample.
